# Ecological Niche Transferability Using Invasive Species as a Case Study

**DOI:** 10.1371/journal.pone.0119891

**Published:** 2015-03-18

**Authors:** Miguel Fernández, Healy Hamilton

**Affiliations:** 1 Department of Integrative Biology, University of California, Berkeley, California, United States of America; 2 German Centre for Integrative Biodiversity Research (iDiv) Halle-Jena-Leipzig, Leipzig, Germany; 3 NatureServe, Arlington, Virginia, United States of America; Shandong University, CHINA

## Abstract

Species distribution modeling is widely applied to predict invasive species distributions and species range shifts under climate change. Accurate predictions depend upon meeting the assumption that ecological niches are conserved, i.e., spatially or temporally transferable. Here we present a multi-taxon comparative analysis of niche conservatism using biological invasion events well documented in natural history museum collections. Our goal is to assess spatial transferability of the climatic niche of a range of noxious terrestrial invasive species using two complementary approaches. First we compare species’ native versus invasive ranges in environmental space using two distinct methods, Principal Components Analysis and Mahalanobis distance. Second we compare species’ native versus invaded ranges in geographic space as estimated using the species distribution modeling technique Maxent and the comparative index Hellinger’s *I*. We find that species exhibit a range of responses, from almost complete transferability, in which the invaded niches completely overlap with the native niches, to a complete dissociation between native and invaded ranges. Intermediate responses included expansion of dimension attributable to either temperature or precipitation derived variables, as well as niche expansion in multiple dimensions. We conclude that the ecological niche in the native range is generally a poor predictor of invaded range and, by analogy, the ecological niche may be a poor predictor of range shifts under climate change. We suggest that assessing dimensions of niche transferability prior to standard species distribution modeling may improve the understanding of species’ dynamics in the invaded range.

## Introduction

Our understanding of species’ spatial distributions is derived from documented records of species’ occurrences in nature. Distributions have been inferred by simply plotting convex hulls around occurrence points on a map, or, with increasing frequency, by applying complex geostatistical tools that integrate specimen location data with fine scale environmental data, resulting in a species distribution model (SDM) [1], also known as an ecological niche model [2], [3]. SDMs have aided researchers in analyzing possible biogeographic scenarios and have provided evocative visualizations; species distribution modeling is now entrenched as one of the more important tools used to simulate species range shifts due to climate change (e.g. [4], [5], [6], [7]) and to predict invasive species range expansions (e.g. [8], [9], [10], [11]).

Despite their wide use and promise [4], [12], these practical summaries of biogeographic information are still controversial [13], [14], [15], [16]. The challenges of applying SDMs fall into four categories: (1) Species data considerations, where the quantity [17], quality [18] and spatial structure [19] of specimen locality data can have profound effects on the model results; (2) Environmental data considerations, where the choice of environmental data [20], [21], grain size [22] and scale [23], [24] can affect the results from SDMs; (3) Issues related to the selection of the SDM technique, where the choice of model algorithm [25], [26], [27], [28], [29] and threshold selection [30] provide high variability among SDM outputs; and (4) Assumptions of species distribution modeling which in some instances are flawed [31], [32], specifically the assumption that biotic influences are unimportant, species genotypic and phenotypic composition is invariable over time and space, and viable populations occur everywhere that environmental conditions are suitable [33], [34], [12].

The first assumption, that biotic influences such as competition, predation and mutualism are not accounted for, has profound consequences when SDMs are applied to predict range shifts due to climate change or predict an invasive species' potential areas of range expansion. The issue of whether biotic interactions will remain similar in a different spatial or temporal context remains a central question in species distribution modeling [4], [35], [36], [37], [38].

The second assumption reflects the inability of SDM to account for the genetic and phenotypic variation among sampled individuals, thus assuming a uniform response to changes in the environment [39], [32]. While in some cases this may be effectively true (e.g. [40]), a large body of evidence describes how founder effect and natural selection in invasive species populations can occur within relatively short time frames, on the order of years [41], [42], [43].

The third assumption, that species occur everywhere suitable conditions exist, suggests that species have had sufficient time and ability to populate all locations of suitable habitat [44], [45]. However, many species are known to not occupy all suitable habitat. Indeed species invasions themselves evidence this, [32] and metapopulations with source-sink dynamics are common [46], [47].

These three assumptions are very convenient when trying to model a species distribution. However, they oversimplify the relationship between species and their environment and whether or not this relationship is maintained in a different temporal or spatial context (i.e., niche transferability) resulting in decreased SDM performance when the model is projected into a different temporal or spatial context (i.e., low transferability). If our goal is to evaluate transferability in predictions on invasive species range expansions, the model should be fitted with data from the native range and predictions should be tested with independent data from the invaded range [13], [48]. Similarly, if the goal is to evaluate transferability in predictions under climate change, the model should be tested against observed range shifts [36], [49], [50]. However, opportunities to conduct these types of analyses are often limited due to the lack of availability of datasets offering spatio/temporal series [51], [39], [32]. Currently, very few SDM studies that project distributions into a different spatial or temporal context provide an evaluation measurement of ecological niche transferability [4], [52]. Here, we evaluate ecological niche transferability, an idea largely neglected [53], [54], [16] with mixed evidence for the occurrence of niche shifts vs. niche conservation [55], [56], [15], [57], [58]. We ask whether invasive species, as geographically documented in natural history museum specimen collections, remain in the same n-dimensional abiotic environmental space when moved into a different spatial context.

## Methods

### Species and geographic occurrence data

Here, we evaluate ecological niche transferability using a subset of 13 species derived from the IUCN’s list of the top 100 Invasive Species [59] known for their large impact on biological diversity or human activities and their illustration of important issues of biological invasion. The subset of species (Table 1) each fulfilled the following criteria: terrestrial macrobiotic organisms, with >100 unique localities in both the native and invaded ranges, downloadable as georeferenced occurrence information (precise to seconds in latitude and longitude) from the Global Biodiversity Information Facility (GBIF). Data points from GBIF were attributed to native or invaded ranges according to range discriminations described in the Global Invasive Species Database. Two geospatial databases were created for each species, one for the native range and one for the invaded range.

**Table 1:** Specimen georeferenced observation data per species from GBIF divided between native and invaded ranges.

Species (English common name)	Native range	Invaded range
*Bufo marinus* (Cane toad)	1175	729
*Cervus elaphus* (Red deer)	9301	224
*Euphorbia esula* (Green spurge)	7655	559
*Lantana camara* (Spanish flag)	814	546
*Leucaena leucocephala* (White leadtree)	391	322
*Linepithema humile* (Argentine ant)	265	202
*Lythrum salicaria* (Purple loosestrife)	54434	752
*Mimosa pigra* (Mimosa)	371	108
*Rana catesbeiana* (American bullfrog)	4141	2187
*Sphagneticola trilobata* (Creeping ox-eye)	324	115
*Sturnus vulgaris* (European starling)	1296	116700
*Tamarix ramosissima* (Saltcedar)	104	416
*Ulex europaeus* (Common gorse)	35241	431

### Environmental layers

The environmental envelopes for each invasive taxon in its native and invaded range was calculated using 19 derived bioclimatic variables (Table 2) acquired from Worldclim 1.4 [60]. We applied a minimum spatial resolution of 1 km^2^ to minimize the effect of small-scale ecological interactions, which define species ranges at scales <1 km^2^ [61], [62].

**Table 2:** List of Worldclim 1.4 environmental variables used in the analysis derived from monthly mean, maximum, and minimum temperature and precipitation interpolations averaged for 1950 to 2000 at a spatial resolution of 1km^2^.

Code	Description
**v1**	Annual Mean Temperature (°C)
**v2**	Mean Diurnal Temp Range (Mean of monthly (max temp—min temp)) (°C)
**v3**	Isothermality (v2/v7) (*100)
**v4**	Temperature Seasonality (standard deviation *100)
**v5**	Max Temperature of Warmest Month (°C)
**v6**	Min Temperature of Coldest Month (°C)
**v7**	Temperature Annual Range (v5–v6) (°C)
**v8**	Mean Temperature of Wettest Quarter (°C)
**v9**	Mean Temperature of Driest Quarter (°C)
**v10**	Mean Temperature of Warmest Quarter (°C)
**v11**	Mean Temperature of Coldest Quarter (°C)
**v12**	Annual Precipitation (mm)
**v13**	Precipitation of Wettest Month (mm)
**v14**	Precipitation of Driest Month (mm)
**v15**	Precipitation Seasonality (Coefficient of Variation)
**v16**	Precipitation of Wettest Quarter (mm)
**v17**	Precipitation of Driest Quarter (mm)
**v18**	Precipitation of Warmest Quarter (mm)
**v19**	Precipitation of Coldest Quarter (mm)

### Comparison of the native and invaded ranges

We compared the native and invaded ranges in two complementary ways: (1) a comparison between observed environmental space occupied by native and invaded populations, and (2) a comparison of the modeled geographic space occupied as predicted using SDM methods. This dual approach dissociates the observed environmental distribution from the geographic distribution estimated using SDM methods, which otherwise are confounded [63], [64].

### Comparison of observed environmental spaces

The range of abiotic environmental parameters at which species were recorded in their native range was compared to those records in the invaded range using two complementary methods. Principal Component Analysis [65], which reduces complex non-parametric multivariate datasets into lower dimensions [66], allows data visualization and also accommodates non-independent datasets as expected in our environmental data. It was performed using SPSS v16.0. The factor analysis rotation method was varimax and factor scores were calculated using the regression method [67]. The three principal components with highest eigenvalues were plotted in three-dimensional space using SigmaPlot v11.0.

Mahalanobis distance [68] is a distance metric based on variable covariance best suited for non-spherically symmetric distributions and deals very robustly with variables with very different scales, such as the 19 bioclimatic variables we used for characterizing niche space [69]. The Mahalanobis distance (MD) between two multivariate vectors $\overset{-}{x}$ and $\overset{-}{y}$ is:

$$MD_{x,y}=\sqrt{{\left(\bar{\bar{x}-\bar{y}}\right)}^{T}S_{x}^{-1}\left(\bar{\bar{x}-\bar{y}}\right)}$$ (1)

in *n-*dimensional space where *S* is the covariance matrix and the suffix *T* denotes that vectors should be transposed. This was calculated using MATLAB v6.5 where the native and invasive groups for each species were randomly permuted one hundred thousand times to produce the underlying probability density. The reported values were based on the mean.

### Comparison of modeled geographic spaces

SDMs were created based on the native and invaded range localities using Maxent v3.3.1 [70], a distribution modeling algorithm many consider to perform better relative to alternative SDM techniques [26], [71]. Standard settings were applied [18] and logistic model outputs were compared using a SDM threshold independent metric that allows the comparison of two continuous raster layers [72]: the modified Hellinger’s *I* statistic,

$$I=1-\frac{1}{2}\sqrt{{\sum (\sqrt{A}-\sqrt{B})}^{2}}$$ (2)

In this statistic, *A* and *B* represents the two SDM outputs that are to be compared pixel by pixel. Statistical significance was assessed following [72], repeating the comparison one hundred times using ENMTools v1.0 software, which creates random samples, with replacement, of the original locality data from the native and invaded ranges. The pseudo-replicate data were then used as input in Maxent to produce a new pair of SDMs. Once the models are created, ENMTools calculates *I* for each new pair of maps and provides an average [72].

## Results

**Fig 1:**
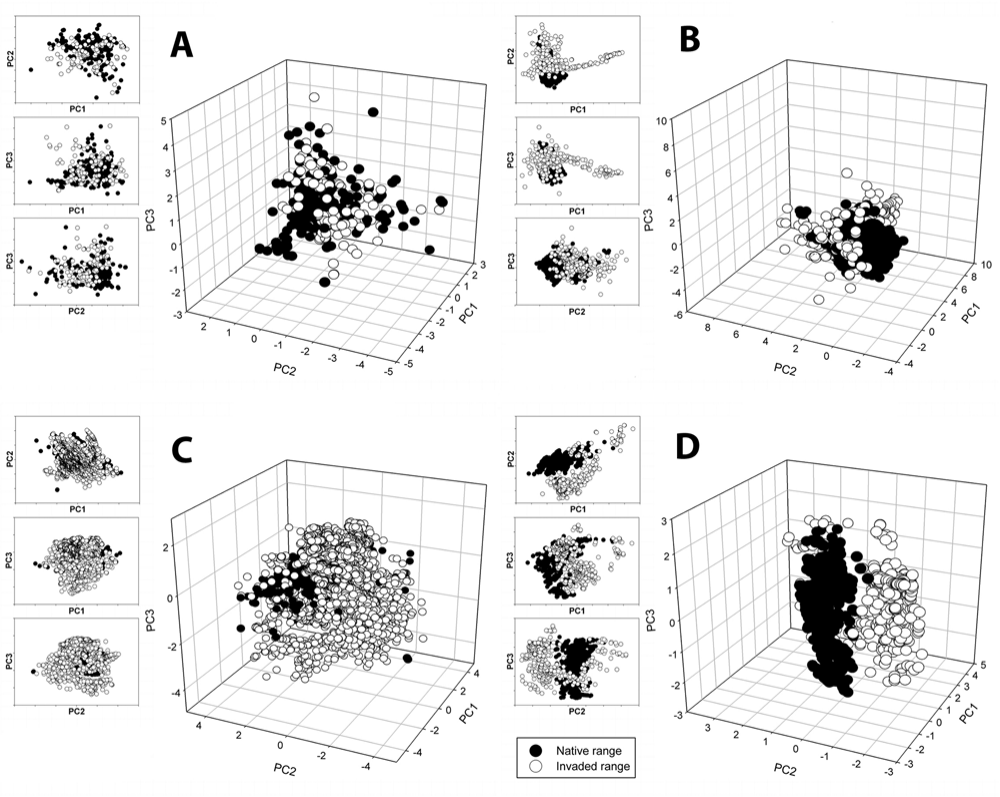
Results from the PCA analysis for: A: *Leucaena leucocephala*, an example of total overlap between native range and invaded range in environmental space. B: *Ulex europaeus*, an example of overlap in environmental space and directional expansion of invaded localities. C: *Sturnus vulgaris*, an example of multidimensional expansion of the invaded environmental space. D: *Rana catesbeiana*, an example of little overlap of the native and invaded environmental space.

The evaluation of ecological niche transferability based on the Principal Component Analysis (PCA) analysis produced four general categories of patterns. The first consisted of complete niche overlap between the native and invaded environmental space (Fig. 1A). For *Bufo marinus*, *Lantana camara*, *Leucaena leucocephala* and *Mimosa pigra*, the environmental space occupied by the species in the native range is almost identical to the environmental space occupied by the invaded range as defined by the PCA.

The second observed pattern consisted of niche expansion in one direction. For *Cervus elaphus*, *Tamarix ramosissima*, *Sphagneticola trilobata* and *Ulex europaeus*, the environmental space occupied by the native range is also shared by the invasive range; however the invasive range also occupies areas of the environmental space that the native does not (Fig. 1B). Importantly, the difference is not in all directions of environmental space, but in only one direction, suggesting that the species is expanding its niche along a few environmental variables while maintaining its native relationship with niche space in others.

The third observed pattern is exhibited by *Sturnus vulgaris*, *Lythrum salicaria* and *Linepithema humile* and reflects a multidirectional expansion of the invaded environmental space in relation to the native niche. These species’ invaded and native clouds overlap, however the invaded range also expands along all three main PCA axes (Fig. 1C).

The final observed pattern is divergence of environmental space. For *Euphorbia esula* and *Rana catesbeiana*, native and invaded environmental spaces show a clear separation (Fig. 1D).

**Fig 2:**
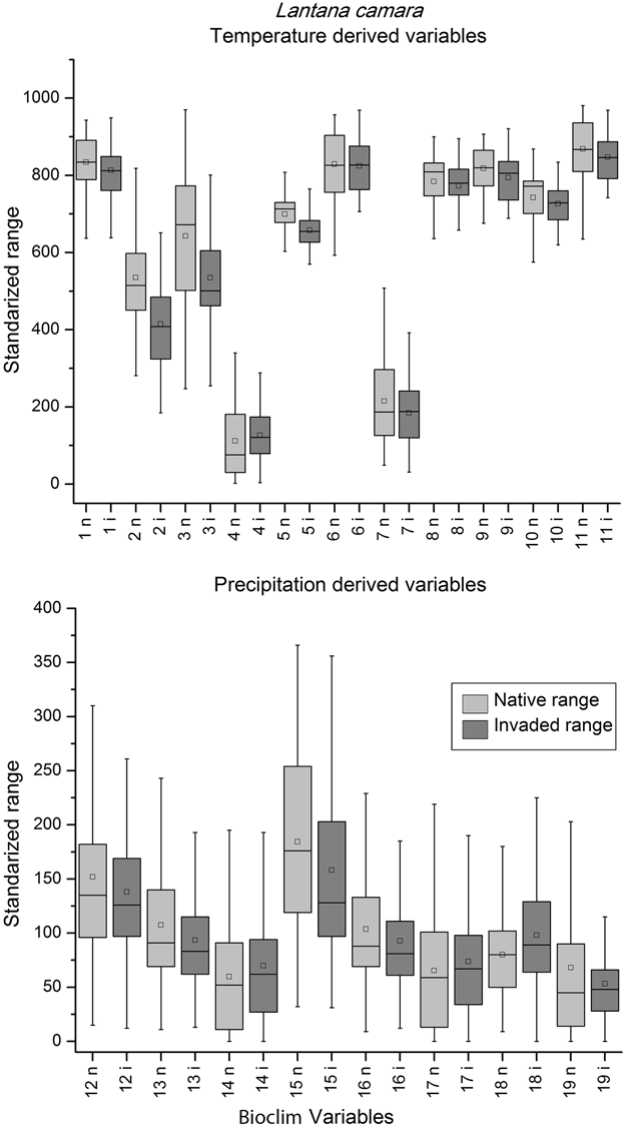
Temperature (top) and precipitation (bottom) derived variables box-plots of extracted native (light grey) and invaded (dark grey) environmental conditions for *Lantana camara*, a species with complete overlap in environmental space. The central rectangle spans the first quartile to the third quartile of the data, the small box represents the mode, the segment inside the rectangle shows the median and "whiskers" above and below the box show the locations of the minimum and maximum values. The numbers in x-axis represent the Bioclim layers.

**Fig 3:**
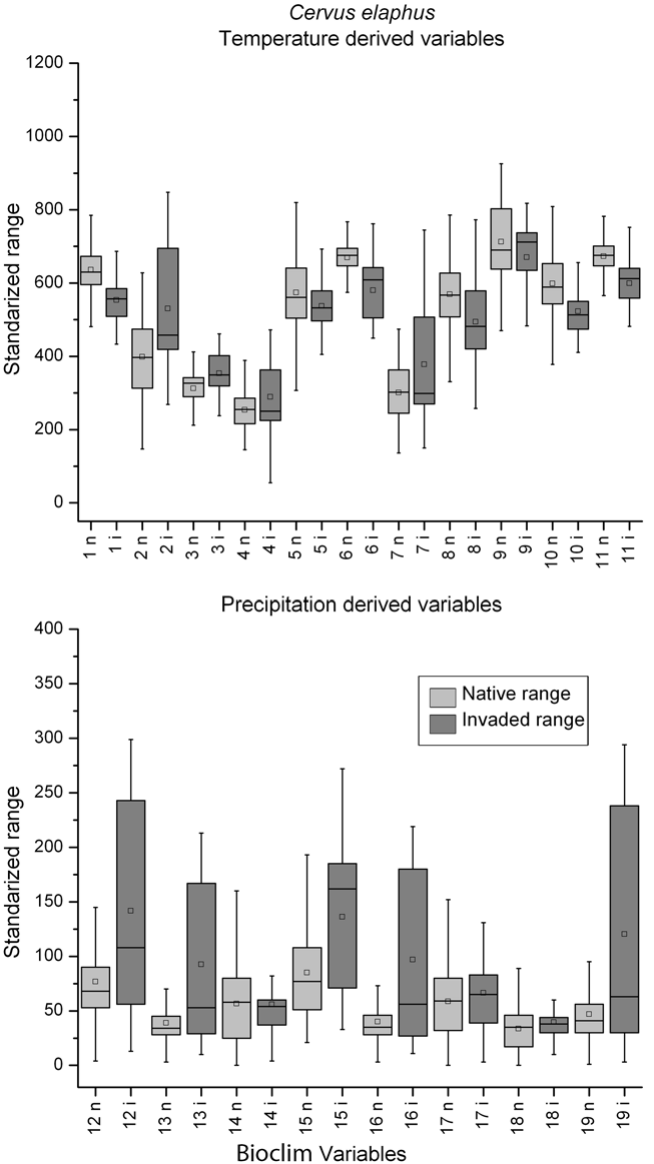
Temperature (top) and precipitation (bottom) derived variables box-plots of extracted native (light grey) and invaded (dark grey) environmental conditions for *Cervus elaphus*, a species with directional expansion. Refer to legend in Fig. 2 for box-plot interpretation.

**Fig 4:**
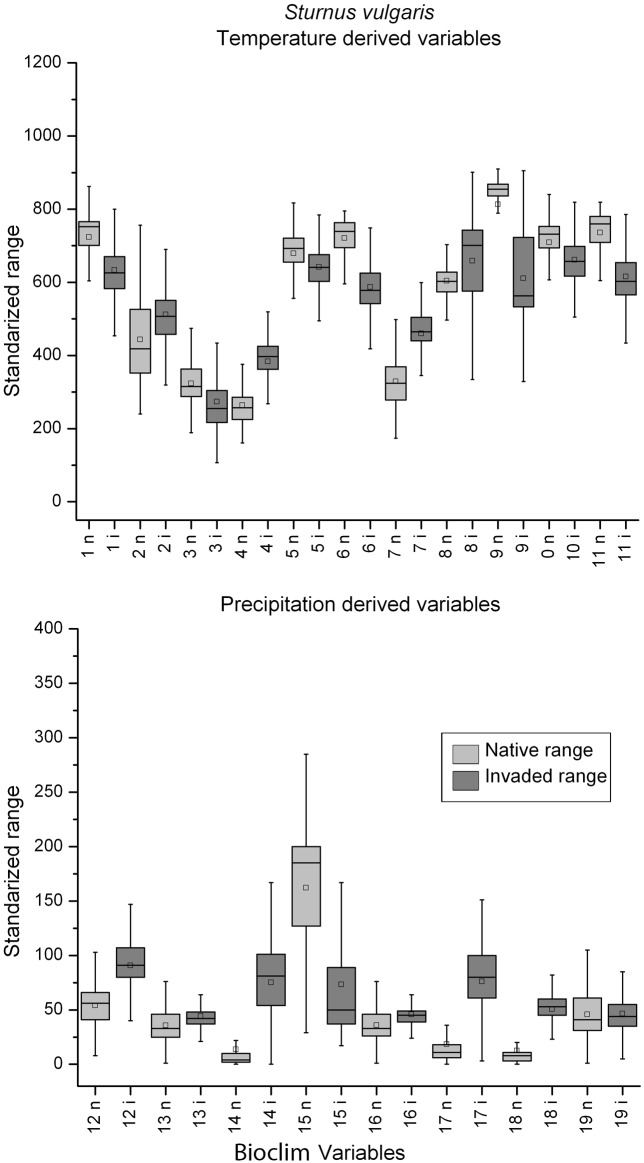
Temperature (top) and precipitation (bottom) derived variables box-plots of extracted native (light grey) and invaded (dark grey) environmental conditions for *Sturnus vulgaris*, a species with multidimensional expansion. Refer to legend in Fig. 2 for box-plot interpretation.

**Fig 5:**
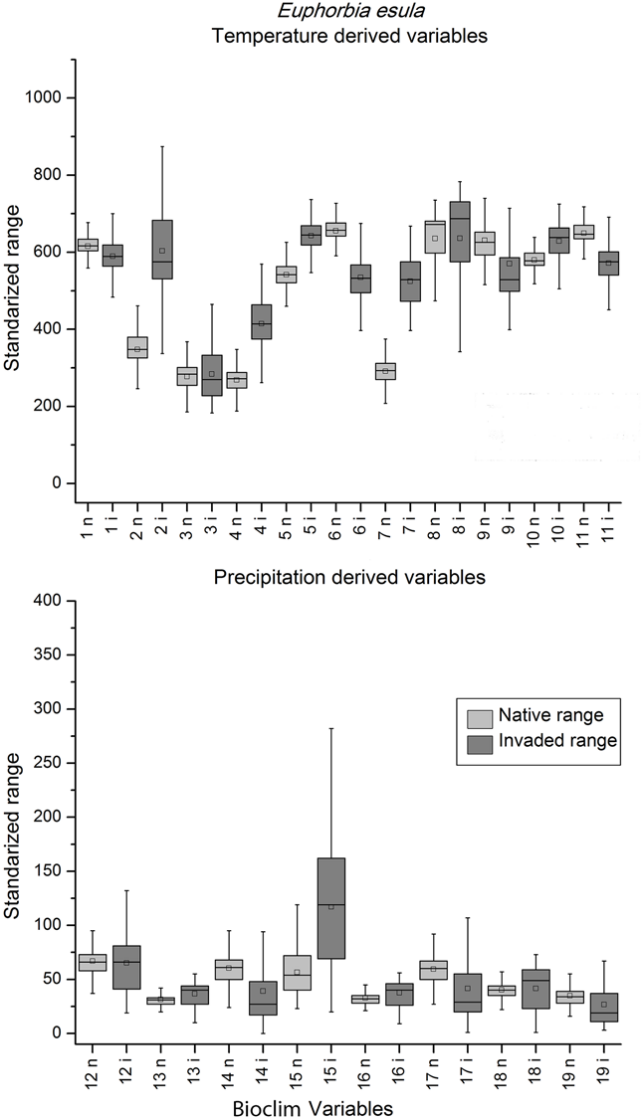
Temperature (top) and precipitation (bottom) derived variables box-plots of extracted native (light grey) and invaded (dark grey) environmental conditions for *Euphorbia esula*, a species with non-overlap in environmental space. Refer to legend in Fig. 2 for box-plot interpretation.

When the box-plots for all variables and species were examined, complete niche overlap was easily distinguishable (Fig. 2). Box-plots of the species with directional expansion showed that the directionality was attributable to either the temperature-derived variables or precipitation, but not both (Fig. 3). The multidirectional expansion and non-overlap patterns were difficult to discern based on the box plots alone (Figs. 4 & 5). In general, environmental differences were more difficult to detect than similarities.

**Fig 6:**
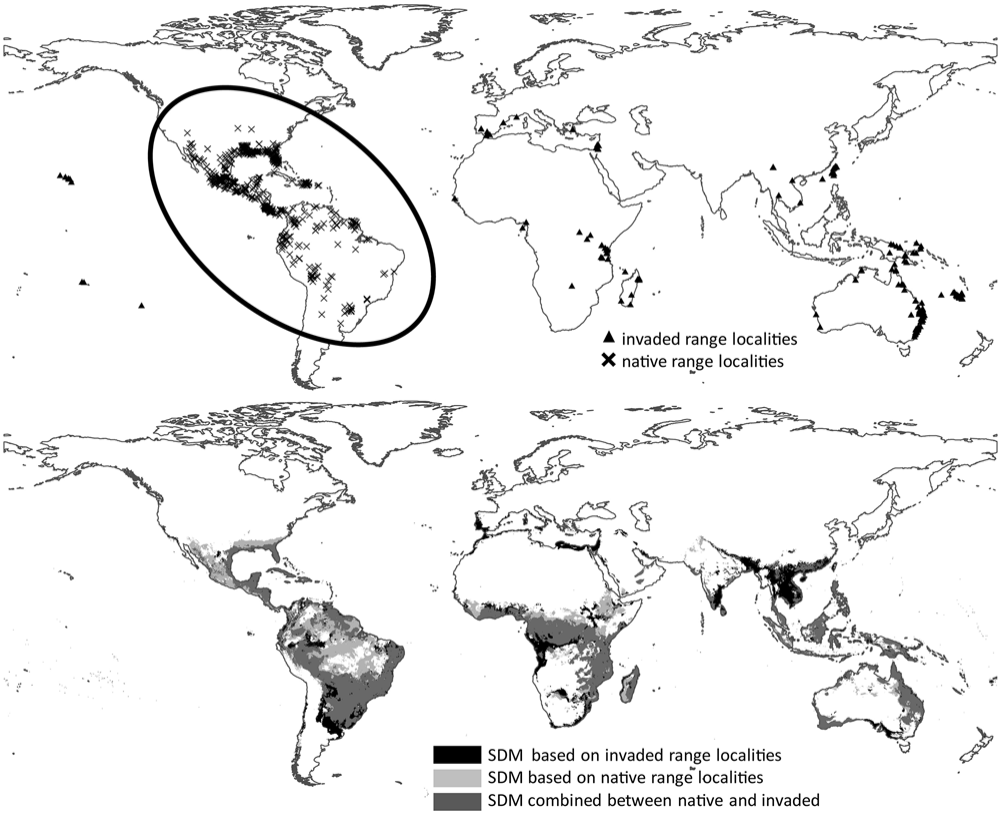
*Lantana camara*. Top: Localities from the native and invaded range (ellipse highlights the native range localities). Bottom: Map of bagging SDM Maxent results. Black shading represents areas predicted to be suitable by only the model trained with invaded range localities. Light grey shading represents areas predicted to be suitable by only the model trained with native range localities. Dark grey shading represents area of overlap between the models trained using native and invaded range localities.

**Fig 7:**
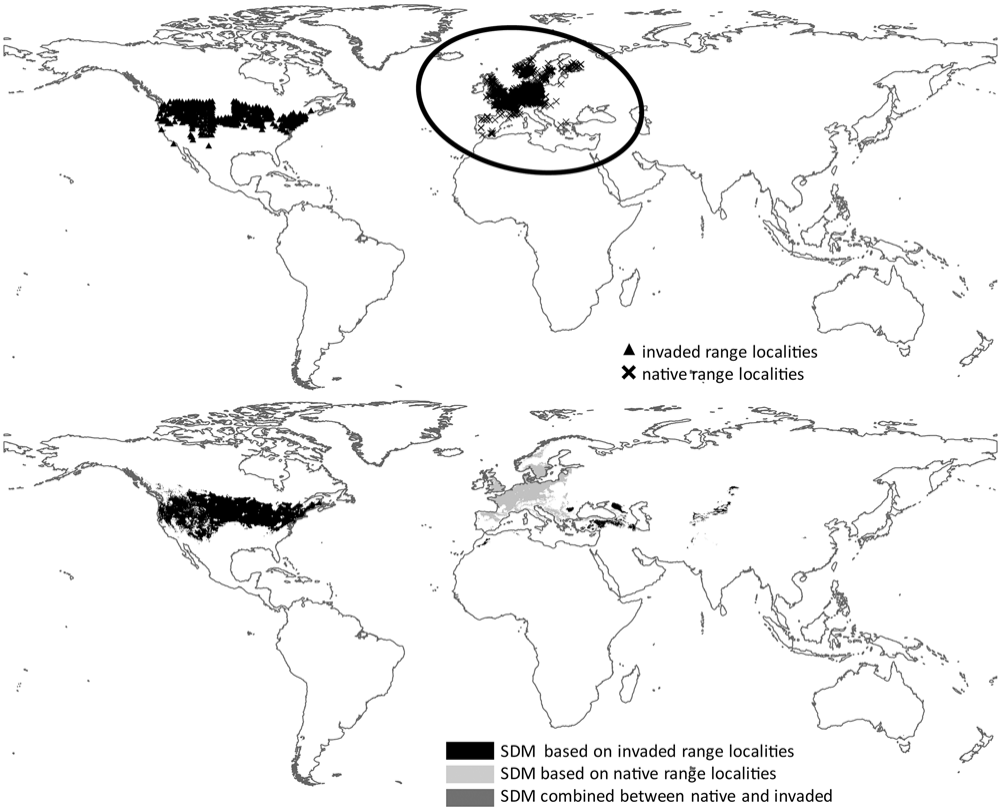
*Euphorbia esula*. Top: Localities from the native and invaded range (ellipse highlights the native range localities). Bottom: Map of bagging SDM Maxent results. Black shading represents areas predicted to be suitable by only the model trained with invaded range localities. Light grey shading represents areas predicted to be suitable by only the model trained with native range localities. Dark grey shading represents area of overlap between the models trained using native and invaded range localities.

Close examination of the maps reveals that species that were ranked very high (i.e. high transferability) by both tests show a high spatial overlap between the native range localities and the SDM Maxent output based on the invaded localities (Fig. 6). The opposite is true for species that were ranked very low (i.e., low transferability) by both tests; these species show almost no spatial overlap between the native range localities and the SDM Maxent output based on the invaded localities (Fig. 7).

**Fig 8:**
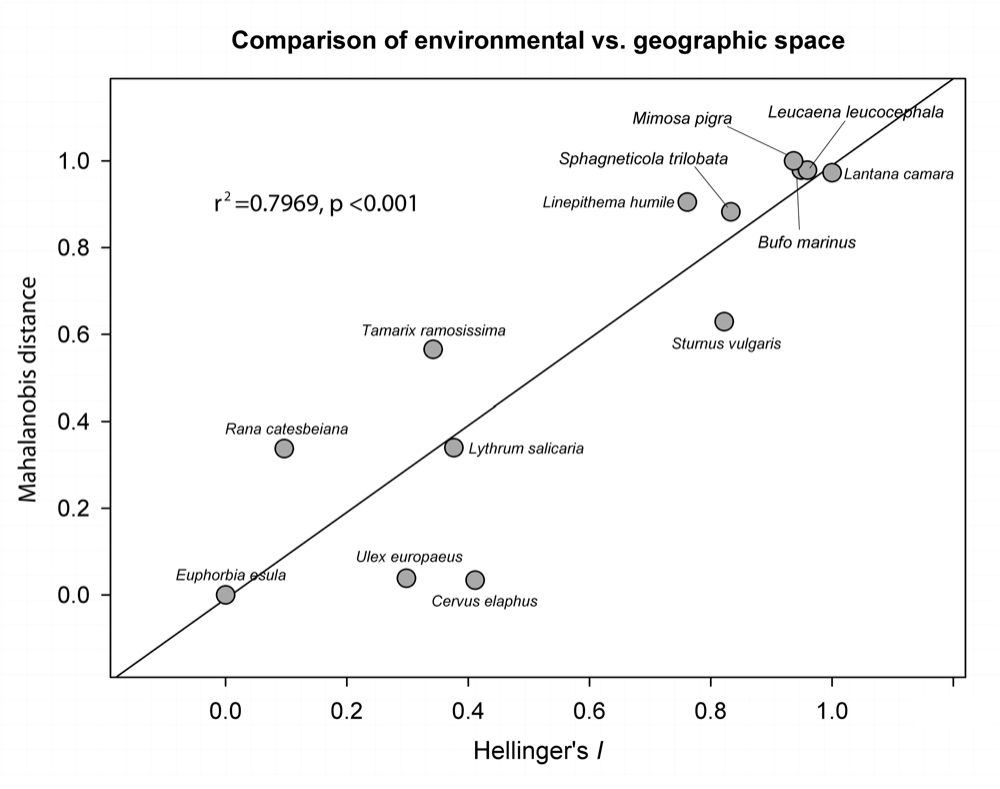
Comparison of environmental vs. geographic space. Comparison of the Mahalanobis distance and the Hellinger's *I* tests distinguishes species with complete overlap between native and invaded range from species, but it does not discern amongst the other three categories where niche space is less conserved. Values were rescaled from 0 to 1; 0 represents low relative similarity and 1 represents high relative similarity.

Finally comparison between the results of the Mahalanobis and Hellinger's *I* test (Fig. 8) showed that both tests ranked the species similarly (r^2^ = 0.80, p <0.001). Species demonstrating total niche overlap in environmental space were discriminated by both tests with similar values.

## Discussion

Invasive species may respond to new environments in various ways, ranging from niche conservatism [73] to low transferability [74]. Our results suggest species responses to new environments can be categorized into four general classes: niche conservatism, niche expansion along specific axes of either temperature- or precipitation-derived variables, niche expansion in multiple dimensions of environmental space, or complete dissociation between native and invaded niches. These results suggest that niche transferability may be a largely species-specific characteristic, although more in-depth studies of particular clades may better resolve correlations between responses and lineages or phenotypes.

While the invasive response of some of these 13 species have already been modeled and analyzed [10], [75], [76], [36], [77], niche transferability has not been assessed in conjunction with standard SDM performance indices. Conclusions about the potential extent of suitable habitat for a newly invaded species based on the environmental conditions of the native range will be compromised if tests of transferability show a lack of niche conservatism. We recommend that niche transferability assessments be considered standard practice in SDMs dealing with invasive species [78].

In our analysis of niche transferability we worked under the assumption that the entire range was limited by climate alone. However, some parts of a species’ distribution can be constrained by climatic factors while other parts can be influenced by biotic interactions [79]. Given that the flora, fauna, and climate of an invaded landscape may be largely if not entirely, novel (to the invader) and heterogenous, we should expect that some species will show different distributions in an invaded region and perhaps different responses in different parts of that range. If a species invades multiple regions, we might also expect its biotic interactions and consequent distributions to be different in each region. In other words, we might expect the distributions of invasive species to show a strong species-by-region interaction effect. Future research should explore the idea that transferability, as a species characteristic, depends on particular geographies under analysis.

Furthermore, interpretation of species’ biogeographic properties may depend on the scale and spatial resolution of analysis [12]. As finer grain size is selected the responsible variables will shift from climatic factors to local disturbances and biotic factors [80]. Our global analysis used one square kilometer grain size as a compromise between file size and data availability; future studies may fruitfully investigate the threshold spatial resolution at which climatic determinants give way to biotic interactions as the strongest correlates defining species range limits.

Multiple statistical tests accompanied by multidimensional visualizations of environmental and geographic space may be required to improve understanding of species dynamics in the invaded range. Many indices of degree of niche similarity (i.e. Kappa, Fuzzy Kappa, Euclidean distance, Schoener *D*, modified Hellinger *I*) have been used in previous research on ecological divergence and speciation [81], [82], [72] as well as SDM uncertainty evaluation [18]. Investigating both approaches—comparing environmental spaces and evaluating geographic projections of environmental space—shows similar but not identical inferences (Fig. 8) and that a single measure of ecological niche transferability may provide a generally accurate but imprecise picture.

Poorly constrained predictions, which complicate translating research results into management strategies, are a common hurdle for resource managers. Since management strategies are often the goal of invasive species modeling research, we offer an example of how our results can be applied directly. *Rana catesbeiana* is native to eastern North America and considered one of the most harmful amphibian invasive species. First, we distinguished native from invaded localities of *R*. *catesbeiana* using the geographic regions designated in [77], [36], then compared environmental space as described in our *Methods*. *R*. *catesbeiana* has very low transferability values (*I* = 0.096, Mahalanobis = 0.338), indicating a clear separation of occupied ecological space between native and invaded ranges. If [77], [36] had evaluated niche transferability, they would have found that *R*. *catesbeiana* has the ability to invade environmental space very unlike that of its native range, thus appropriately reducing confidence in predictions of their SDM. This clearer understanding of the dynamics of *R*. *catesbeiana* in its invaded range emphasizes the importance of control programs preventing transport from source locations, since invaded locations cannot necessarily be predicted from native distributions. Testing for niche transferability can help inform the appropriate use of SDM in modeling biological invasions and thereby improve invasion management strategies.
